# Clinical and Metabolomic Effects of *Lactiplantibacillus* *plantarum* and *Pediococcus acidilactici* in Fructose Intolerant Patients

**DOI:** 10.3390/nu14122488

**Published:** 2022-06-15

**Authors:** Piero Portincasa, Giuseppe Celano, Nadia Serale, Paola Vitellio, Francesco Maria Calabrese, Alexandra Chira, Liliana David, Dan L. Dumitrascu, Maria De Angelis

**Affiliations:** 1 Clinica Medica “A. Murri”, Department of Biomedical Sciences & Human Oncology, University of Bari Medical School, 70124 Bari, Italy; 2 Dipartimento di Scienze del Suolo, della Pianta e Degli Alimenti, Università Degli Studi di Bari Aldo Moro, 70126 Bari, Italy; giuseppe.celano@uniba.it (G.C.); nadia.serale@uniba.it (N.S.); paolavitellio91@gmail.com (P.V.); francesco.calabrese@uniba.it (F.M.C.); 3 2nd Department of Internal Medicine, ‘Iuliu Hatieganu’ University of Medicine and Farmacy, 400012 Cluj-Napoca, Romania; achira@umfcluj.ro (A.C.); ldavid@umfcluj.ro (L.D.)

**Keywords:** functional gastrointestinal disorders, intestinal barrier, fructose intolerance, metabolome, short-chain fatty acids

## Abstract

Fructose intolerance (FI) is a widespread non-genetic condition in which the incomplete absorption of fructose leads to gastro-intestinal disorders. The crucial role of microbial dysbiosis on the onset of these intolerance symptoms together with their persistence under free fructose diets are driving the scientific community towards the use of probiotics as a novel therapeutic approach. In this study, we evaluated the prevalence of FI in a cohort composed of Romanian adults with Functional Grastrointestinal Disorders (FGIDs) and the effectiveness of treatment based on the probiotic formulation EQBIOTA^®^ (*Lactiplantibacillus plantarum* CECT 7484 and 7485 and *Pediococcus acidilactici* CECT 7483). We evaluated the impact of a 30-day treatment both on FI subjects and healthy volunteers. The gastrointestinal symptoms and fecal volatile metabolome were evaluated. A statistically significant improvement of symptoms (i.e., bloating, and abdominal pain) was reported in FI patient after treatment. On the other hand, at the baseline, the content of volatile metabolites was heterogeneously distributed between the two study arms, whereas the treatment led differences to decrease. From our analysis, how some metabolomics compounds were correlated with the improvement and worsening of clinical symptoms clearly emerged. Preliminary observations suggested how the improvement of gastrointestinal symptoms could be induced by the increase of anti-inflammatory and protective substrates. A deeper investigation in a larger patient cohort subjected to a prolonged treatment would allow a more comprehensive evaluation of the probiotic treatment effects.

## 1. Introduction

Functional Gastrointestinal Disorders (FGIDs) are globally recognized to be the most commonly identified gastrointestinal disorders. In addition to a significant impairment of life quality and psychological well-being, FGIDs considerably impact direct costs on patients and on the whole health-care system, as well on secondary costs due to reduced workforce productivity and absenteeism [1].

Several factors contribute to the genesis of FGIDs, and include intestinal dysbiosis [2], genetic predisposition, symptom perception, foods, lifestyle, and intestinal dysmotility [3]. In addition, FGIDs are often associated with food intolerance toward several Fermentable Oligosaccharides, Disaccharides, Monosaccharides and Polyols (FODMAPs). Food intolerances are, in fact, adverse reactions to food or happening after single ingredient exposure [4]. Most frequent symptoms include bloating, abdominal pain, flatulence, discomfort, diarrhea, and/or constipation [5].

**Figure 1:**
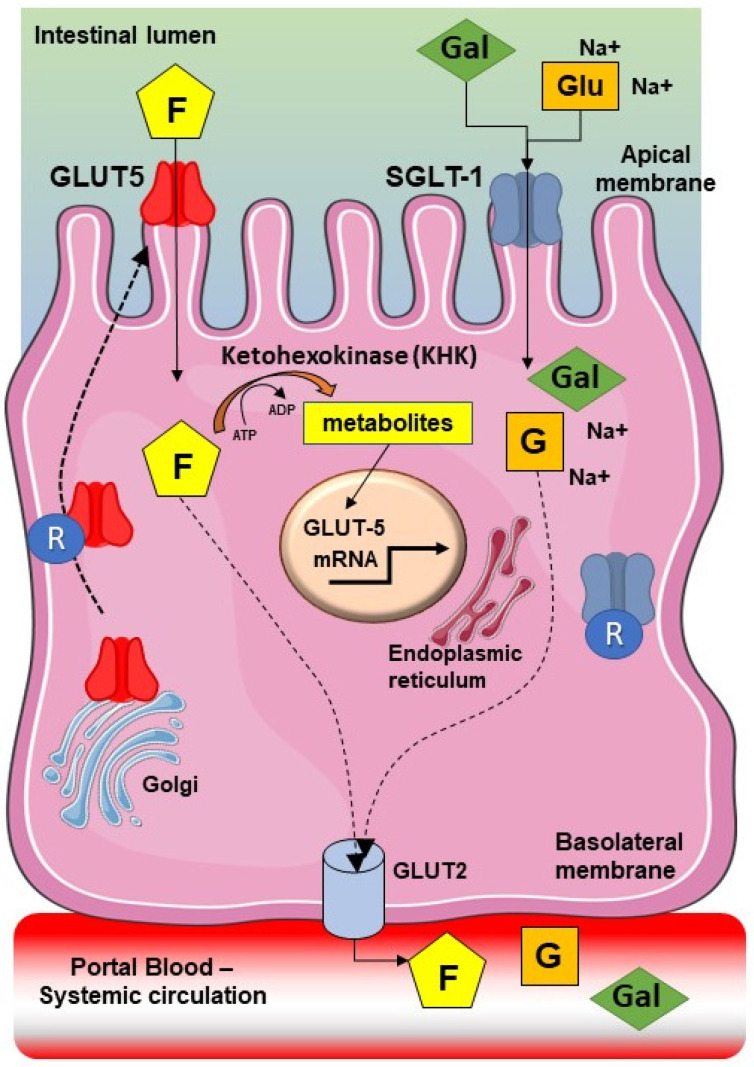
Steps involved in intestinal fructose transport across the small intestinal epithelia. Fructose (F) from diet is transported in monosaccharaide form across the apical membrane by the facilitative glucose transporter 5 (GLUT5, *Slc2a5*), a member of the facilitative GLUT family (gene family *Slc2a*). Glucose (Glu) or galactose (Gal) enter the enterocyte via the apical membrane via the Na+-dependent glucose transporter 1 (SGLT2, *Slc2a2*) and Na+-coupled cotransport. Some fructose is phosphorylated by ketohexokinase (KHK), and this step keeps the lumen-to-cytosol gradient favorable for fructose uptake. In the presence of high luminal fructose concentrations, fructose metabolites stimulate the transcription and translation of GLUT5 and fructolytic enzymes. From the cytosol, most fructose enters the portal vein via the basolateral GLUT2 which also transports glucose (G) and galactose. GLUT2 has a K_m_ for fructose more than fivefold higher than that of GLUT5, (R), receptor. If luminal fructose levels are high, the newly synthesized GLUT5 is delivered to the apical membrane via Rab11a (R), the Ras-related protein-in-brain 11 (Rabil). Rab11a is a small guanosine 5′-triphosphatase that is associated with recycling endosomes and plays a key role in the trafficking of various proteins to the apical membrane. This step increases the transapical fructose transport [13,15].

Found in many plants, fructose, a 6-carbon polyhydroxyketone, is often bonded to glucose in order to form the sucrose disaccharide. Fructose belongs to the family of free or short-chain carbohydrates FODMAPs, and together with glucose and galactose, is one of the three dietary monosaccharides which is passed directly into blood during digestion. On a clinical perspective, the term fructose intolerance (FI) depends on the incomplete intestinal absorption of the monosaccharide fructose, with associated gastrointestinal symptoms, including abdominal pain, bloating, and diarrhea [2]. FI differs from simple fructose malabsorption which is characterized by an inadequate intestinal ability in transporting fructose through the epithelium without causing symptoms [6]. The estimated prevalence of fructose malabsorption in healthy adults is ~34% and can reach higher levels in patients affected by FGIDs [7,8]. Fructose is usually found in natural foods such as fruit and honey. Since fructose is the sweetest sugar, it is industrially used as a sweetener in many processed foods and drinks [9]. Fructose, free or bound, accounts for about half of sweetener food additives [10]. The use of caloric sweeteners is widespread in USA and Europe, where it accounts for 20% of total energy intake. In the western diet, according to the US Department of Agriculture (USDA), fructose consumption has increased by more than 1000% between 1970 and 1990 [11], and more than one-third from 1978 to 2004 [12]. When ingested, fructose is absorbed in the intestine via the apical glucose transporters GLUT5 (main transporter) and GLUT2 in the basolateral membrane [13]. GLUT-5 is induced by increased fructose intake via transcriptional activation. In addition, GLUT5 is dependent upon the presence of KHK- and Rab11a-mediated endosomal protein trafficking [14] (Figure 1).

Of note, chronic feeding, acute gavage-feeding, and in vivo intestinal perfusion of fructose increase GLUT5 expression and activity that occur primarily in the proximal regions of human and rodent small intestine [13,15]. After absorption, fructose is released into the portal bloodstream and in the liver is metabolized to glucose, lactate, and glycogen [16].

Since fructose absorption capacity is limited, just small quantities of sugar are absorbed, whereas the excess in the intestinal lumen causes an osmotic load that draws the fluid. This causes a distention of the small intestine and leading to gastrointestinal symptoms [9]. Furthermore, in mice a high fructose diet induces insulin resistance, dyslipidemia, glucose intolerance, and inflammation [17]. Worsening of clinical symptoms in FI has also been correlated with the occurrence of dysbiosis and with the production of metabolites and gases [18]. This laid the groundwork to the potential role of microbial dysbiosis in the onset of symptoms [6].

Microbial gut dysbiosis may be involved in the onset and maintenance of chronic conditions, such as FGIDs and food intolerances [19]. Indeed, the metabolism associated with the gut microbiota can strongly affect host health status. In normal conditions, gut microbiota produces 50–100 mmol·L^−1^ per day of metabolites, including esters, aldehydes, and short-chain fatty acids (SCFAs), such as acetic, propionic, and butyric acids, that represent an energy source to the host intestinal *epithelium* [20,21,22]. Metabolic activities of gut microbiota, especially in gut dysbiosis, can also result in the overproduction of harmful substances, involved in the pathogenesis and inflammation (hydrogen sulphide, *p*-cresol, and indoxyl sulfate). In addition, dysbiosis induces gut permeability for bacterial harmful products and it increases the exposure to injurious substances that contribute to inflammation and symptom severity [23,24,25,26].

Although lactose and fructose intolerances have been recognized for a long time, their managements continue to be discussed. To prevent symptoms of FI, one of the most used treatment concerns fructose dietary restrictions [27]. Although free fructose diet strongly influences fructose malabsorption, symptoms can persist. Furthermore, the elimination of fructose from the diet might cause the deprivation of healthy foods, such as fruits and vegetables [2]. Therefore, an adequate diet regimen is not the only approach useful in treating these disorders. Nowadays, the effectiveness of probiotics has gained much more interest as potential treatment in FI [28]. Probiotics are live micro-organisms which, when administered in adequate amounts, confer health benefits on the host [29]. It is well known that probiotic strains have positive effects by improving gastrointestinal symptoms correlated with FGID and intolerances [30,31]. Probiotics can also reinforce the intestinal mucosal barrier and normalize the gut permeability and motility and its visceral sensitivity [32]. A recent review highlighted the beneficial effects of probiotics on lactose intolerance symptoms, such as the reduction of abdominal cramps, diarrhea, vomiting, bloating, and flatulence [30]. Other studies reported that *Lactiplantibacillus plantarum* strains are known for their antimicrobial proprieties in treating dysbiosis in FGIDs [33,34]. The combination of *L. plantarum* and *Pediococcus acidilactici* has been demonstrated to have a positive effect in the decrease in low-density lipoprotein (LDL) cholesterol [35] and was proven to exert clinical benefits on food intolerances and correlated obesity [36,37].

Thus, the aim of this study was to assess (i) the prevalence of fructose intolerance in patients with FGIDs; (ii) the effectiveness of 30-days-treatment with a novel probiotic formulation, i.e., EQBIOTA^®^ (*L. plantarum*—CECT 7484 e 7485 strains—and *P. acidilactici* CECT 7483) in improving symptoms in patients with fructose intolerance on a fructose-free diet regimen and persistent symptomatology.

## 2. Materials and Methods

### 2.1. Cohort of Patients and Recruitment Criteria

An initial cohort of 69 Romanian adult patients (mean age 53 ± 15 SD years, 26 males and 43 females) with FGIDs were initially included in this study. Patients were seen at the outpatient clinic of the 2nd Medical Clinic of the University hospital in Cluj-Napoca, Romania, which is a referral center for gastrointestinal symptoms. Exclusion criteria for the subject enrolment were: evidence of small intestinal bacterial overgrowth (SIBO) according to standard criteria [38] and/or organic disease, occurrence of inflammatory bowel diseases, occurrence of cardiovascular diseases, liver and kidney diseases, psychiatric disorders, and pregnancy. Subjects with a diagnosis of irritable bowel syndrome (IBS), according to Rome IV criteria, under treatment with nonsteroidal anti-inflammatory drugs, anticoagulants, or antibiotics were also excluded. Fibers or probiotics, alcohol beverages, and treatments acting on intestinal motility were not allowed in the 4 weeks before and during the study.

### 2.2. Lactose and Fructose Breath Test

The cohort of patients enrolled in the study underwent the fructose and lactose breath test. No antibiotics, colonoscopy, or laxatives were permitted within 14 days and a specific low-saccharide diet was adhered to one day before the tests. Patients arrived for testing in the morning, after fasting overnight. Smoking and physical exercise were not permitted during the day before the test. Lactose and/or fructose malabsorption was defined as an increase ≥20 parts per million (ppm) without symptoms [38]. Intolerance was defined as an increase ≥20 ppm over the baseline associated with symptoms such as abdominal distension or bloating, flatulence, fullness, nausea, diarrhea, and abdominal cramps within the 180 min of observation time. The fermentable substrates used for the lactose and fructose hydrogen breath test were lactose (500 mL of cow’s fresh whole milk containing 25 g) or fructose (500 mL of cow’s fresh whole milk containing 35 g). The operator measured the time-dependent concentrations of H2 in breath samples with an automatic portable analyzer (Gastro + Gastrolyzer^®^, Kent, England), characterized by an accuracy of ±2 ppm, a resolution of 1 ppm, and a range of 0–500 ppm. Lactose-intolerant patients were excluded, while the remaining fructose-intolerant patients were enrolled in the protocol.

### 2.3. Probiotic Treatment with EQBIOTA

All patients positive to fructose intolerance underwent a fructose- and sorbitol-free diet for 30 days. All subjects who did not report an improvement of symptoms after diet were enrolled in the trial and started the treatment with 1 capsule per day of EQBIOTA^®^ (*L. plantarum* CECT 7484 one billion CFU, *L. plantarum* CECT 748 one billion CFU, and *P. acidilactici* CECT 7483 one billion CFU based on cell survival assays). The EQBIOTA probiotic is a food supplement with lactobacilli (beneficial bacteria). Normally, EQBIOTA is taken to promote the balance of intestinal microbiota and maintain normal intestinal function.

As control, 14 sex- and age-matched healthy lean subjects were recruited and underwent treatment. In this group, one subject dropped-out.

After enrolment, patients entered a program including run-in period (30 days) and treatment (30 days). Evaluation of symptoms and sample collection were performed at two different time points, before (T0) and after treatment (T30). This study was performed in accordance with the Helsinki Declaration. The Ethics Committee provided the approval for the study in Cluj-Napoca Hospital (protocol number 7711032019).

### 2.4. Clinical Features and Questionnaires

Patients were evaluated for intensity of symptoms (abdominal pain and bloating), graded on a Visual Analogue Scale (VAS) ranging from 0 to 100 mm. In addition, changes in stool consistency related to mealtimes were documented. Bowel habits were assessed by the Bristol Stool Form Scale (BSFS) [39]. This scale represents a diagnosis instrument for classifying stool (1–7) and thus bowel habits. In particular: Type 1–2 indicate constipation; Type 3–4 are associated with normal stool; Type 5–7 indicate diarrhea. Lifestyle and dietary habits were assessed using the MED-style questionnaire [2,40].

### 2.5. Fecal Collection

Subjects (fructose intolerants and healthy controls) provided fecal samples at the two study time points. After collection, feces were stored as previously described [41] at −80 °C for metabolomics analyses.

### 2.6. Fecal Metabolome

One gram of fecal sample was placed into 10 mL glass vials and added to 10 μL of 4-methyl-2-pentanol (final concentration of 33 mg/L) as the internal standard. Samples were equilibrated for 10 min at 60 °C. SPME fiber (divinylbenzene/Carboxen/polydimethylsiloxane) was exposed to each sample for 40 min. The Volatile Organic Compounds (VOCs) were thermally desorbed by immediately transferring the fiber into the heated injection port (220 °C) of a Clarus 680 (Perkin Elmer, Beaconsfield, UK) gas chromatography equipped with an Rtx-WAX column (30 m × 0.25 mm i.d., 0.25 μm film thickness) (Restek) and coupled to a Clarus SQ8MS (Perkin Elmer). The column temperature was set initially at 35 °C for 8 min, then increased to 60 °C at 4 °C min^−1^, to 160 °C at 6 °C min^−1^, and finally to 200 °C at 20 °C min^−1^ and held for 15 min. Electron ionization masses were recorded at 70 eV in the mass-to-charge ratio interval, which was *m*/*z* 34 to 350. The Gas Chromatography–Mass Spectrometry (GC-MS) generated a chromatogram with peaks representing individual compounds. Each chromatogram was analyzed for peak identification using the National Institute of Standard and Technology 2008 (NIST) library. A peak area threshold of >1,000,000 and 85% or greater probability of match was used for VOC identification, followed by manual visual inspection of the fragment patterns when required. 4-methyl-2-pentanol (final concentration 33 mg/L) was used as an internal standard in all analyses, to quantify the identified compounds by interpolation of the relative areas versus the internal standard area.

### 2.7. Outcomes

The primary outcome of this study was the evaluation of the effect of EQBIOTA^®^ treatment (30 days) on gastrointestinal symptom in patients with persisting symptoms after fructose/sorbitol-free diet, compared with healthy subjects.

### 2.8. Statistical Analysis

The sample size was calculated assuming a 35% difference in response between treatment in fructose-intolerant subjects and healthy control. We estimated that 13 patients would be required for the study to have 75% power and an α error of 5%. Statistically significant differences in clinical symptoms and volatile organic compounds were detected by the Wilcoxon or Mann–Whitney test. Analyses were performed with GraphPad Prism 8.0 (GraphPad Software, Inc., San Diego, CA, USA) and Statistica 7.0 for Windows. Differences between groups were considered significant at a *p*-value < 0.05. Correlations analyses between the amount of volatile organic compounds and clinical symptoms (*p*-value < 0.05) were assessed based on Spearman’s correlation and the results were graphically rendered using GraphPad Prism 8.0 and SigmaPlot 14.5 (Slough Inpixon, UK). Metabolomics data were subjected to permutation analysis using PermutMatrix.

## 3. Results

### 3.1. Prevalence of Fructose Intolerance in FGIDs Cohort and Baseline Characteristics

**Figure 2:**
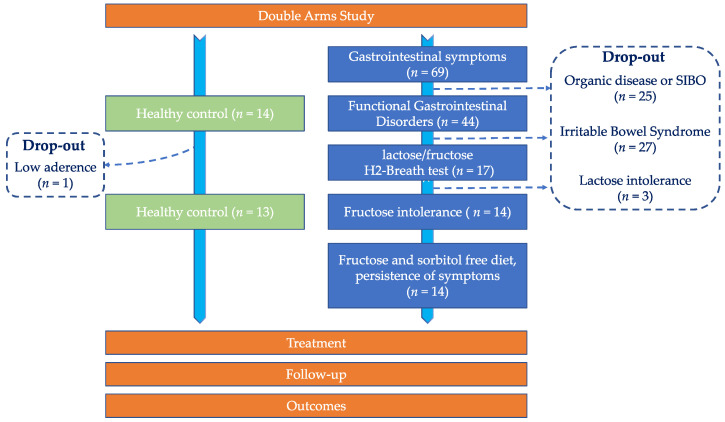
Consort flow-chart of screened patients. Starting from a group of patients with gastrointestinal symptoms, the subjects were screened according to exclusion criteria and underwent a lactose/fructose H_2_ breath test. The fructose intolerant subjects followed a sorbitol and fructose-free diet and those with persistence of symptomatology were enrolled. As control group, 14 healthy subjects (one dropped out) were enrolled. The enrolled subjects underwent a 30-days treatment with EQBIOTA.

From the initial cohort, 25 patients did not meet the inclusion criteria since they were diagnosed with SIBO or were excluded due to the concomitance of organic diseases (Figure 2). Patients with a diagnosis of IBS were also excluded (*n* = 27), according to the Rome IV criteria. The 17 remaining patients underwent the hydrogen (H_2_) breath test to detect fructose or lactose intolerance (LI). Three patients (6.8% of the 44 FGIDs subjects considered) were lactose intolerant, while 14 patients (31.8% of total FGIDs patients) were purely fructose intolerant. In fact, none of the subjects reported the co-occurrence of both fructose and lactose intolerance. Thus, the final group of enrolled patients consisted of 14 fructose-intolerant subjects. Within the control group, one subjects dropped out, and thus 13 healthy subjects (HC) were eligible for the EQBIOTA treatment.

The baseline characteristics of the two cohorts (FI and HC) according to age, sex, body mass index (BMI), symptoms, and bowel habits appear in Table 1. The female sex percentage in HC and FI groups was 46% and 57%, respectively (n.s.). On average, the subjects considered were overweight; in fact, the mean BMI was 28.4 Kg/m^2^ and 25.7 Kg/m^2^ in HC and FI, respectively. Of the 13 healthy subjects, 5 were normal weight, 4 overweight, and 4 obese. Within FI subjects, one was underweight, six were normal weight, three overweight, and four obese. In this group, the prevalence of subjects showing Bristol scores out of normal range (i.e., 3–4) was 50%. In detail, 43% of subjects were constipated and 7% showed a diarrheic bowel habit. Based on clinical features, the two groups at the baseline showed statistically significant differences in term of bloating and abdominal pain (Table 1). In detail, in FI group, the values of gastro-intestinal symptoms (VAS) were significantly higher than HC.

### 3.2. Diet

BMI remained stable throughout the observation period in both HC and FI. Dietary fiber intake was lower than the recommended amount of daily fiber (adults = 12.6–16.7 g/1000 Kcal, SINU 2014). The daily carbohydrate intake fulfilled the reference range for macronutrients (45–60% daily calorie intake, SINU 2014). The protein intake was in the reference range of 15–20%. The percentage of lipids taken daily was in the limits of that recommended (20–35% of the total calories introduced with the diet) with a percentage of saturated fatty acids higher than that established by the guidelines as a nutritional goal, useful for the prevention of cardiovascular diseases (<10% of daily caloric intake, SINU 2014). The daily amount of vitamin B6 was comparable to the average requirement that is 1.1 mg/day (1.15 ± 0.2). The intake of micro- and macro-nutrients was similar in HC and FI subjects (Table 2); therefore, the diet did not impact symptom variation.

### 3.3. Clinical Scores

**Figure 3:**
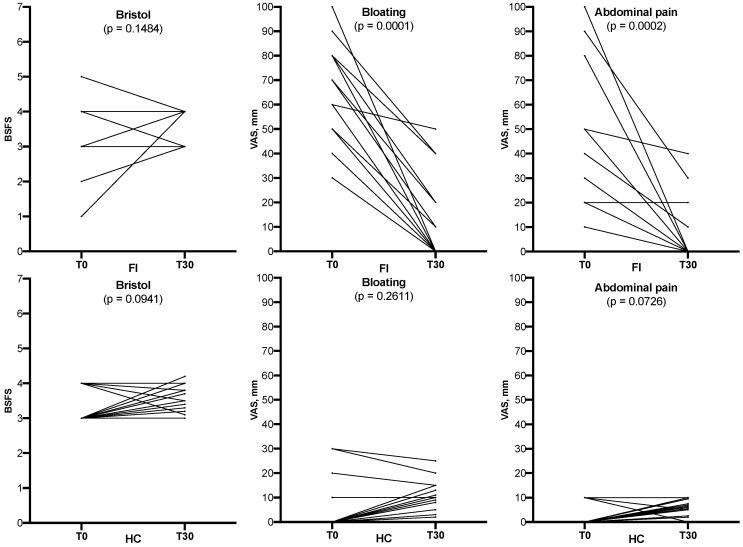
Representation of bowel habits (Bristol Stool Form Scale, BSFS), bloating, and abdominal pain (Visual Analogue Scale, VAS, 0–100 mm) in 14 fructose-intolerant (FI) patients and in 13 healthy controls (HC) at baseline (T0) and after 30 days (T30) of treatment.

Enrolled subjects were treated with EQBIOTA for 30 days and the clinical features were collected. The BMI remained stable throughout the observation period, as well as the intake of micro- and macro-nutrients. Individual changes of symptoms are depicted in Figure 3 and showed an overall improvement of symptoms in FI subjects. In this group, a high percentage (46%) of subjects reported a low Bristol score (constipation score 1–2). However, all patients showing at the baseline Bristol score values out of the “normal” range reached normal values after treatment. Although the globally assessed bowel habits (Bristol score) did not significantly differ after treatment, we observed a significant decrease of bloating (*p* = 0.0001) and abdominal pain (*p* = 0.0002) when compared with baseline (Table 3). Within the healthy control group, no statistically significant differences were detected by comparing T0 and T30 (Table 3, Figure 3).

### 3.4. Probiotics Affects the Fecal Metabolome of FI Subjects

An objective comparison of metabolomic profile in fecal samples from HC and FI groups at the baseline (T0) and after 30 days of treatment (T30) with EQBIOTA^®^ was performed based on qualitative and quantitative differences in VOCs using HS-SPME GC–MS methodology. One hundred and eighteen volatile compounds were identified and grouped according to chemical classes, i.e., alcohols (15), esters (36), aldehydes (4), phenols (5), ketones (9), organic acids (11), terpenes (15), hydrocarbons (19), indoles (2), and sulfur compounds (2) (Supplementary Table S1). Some significant differences were evaluated by comparing HC and FI groups at the T0 (1-pentanol, 3-phenylpropanol, 3-methyl-butanal, formic acid butyl ester, cyclohexanecarboxylic acid butyl ester, cetene, and 2-amino-4-methoxyphenol) and T30 (9-octadecen-1-ol, 2-tridecanol, phenol, 2,4-bis(1,1-dimethylethyl), 6-pentadecen-1-ol, 6,11-dimethyl-2,6,10-dodecatrin-1-ol, eicosane, gamma-Dodecalactone, 2-tridecanone, 1-(2-aminophenyl)-ethanone, heptanoic acid, alfa-humulene, and beta-selinene) (Table 4). Moreover, some statistically significant differences were found after the treatment in the FI group. Compared with baseline, a higher amount of alcohols (1-pentanol and sulcatol), organic acids (hexanoic acid and heptanoic acid), hexadecanal, hexadecane, squalene, and carvacrol was evaluated in fecal samples of FI after 30 days of EQBIOTA treatment. Moreover, this group (FI-T30) was characterized by a lower amount (*p* < 0.05) of 3-methyl-butanoic acid. Concerning the HC group, a higher concentration (*p* < 0.05) of phenylethyl alcohol was assessed in the HC-treated group compared with the baseline.

**Figure 4:**
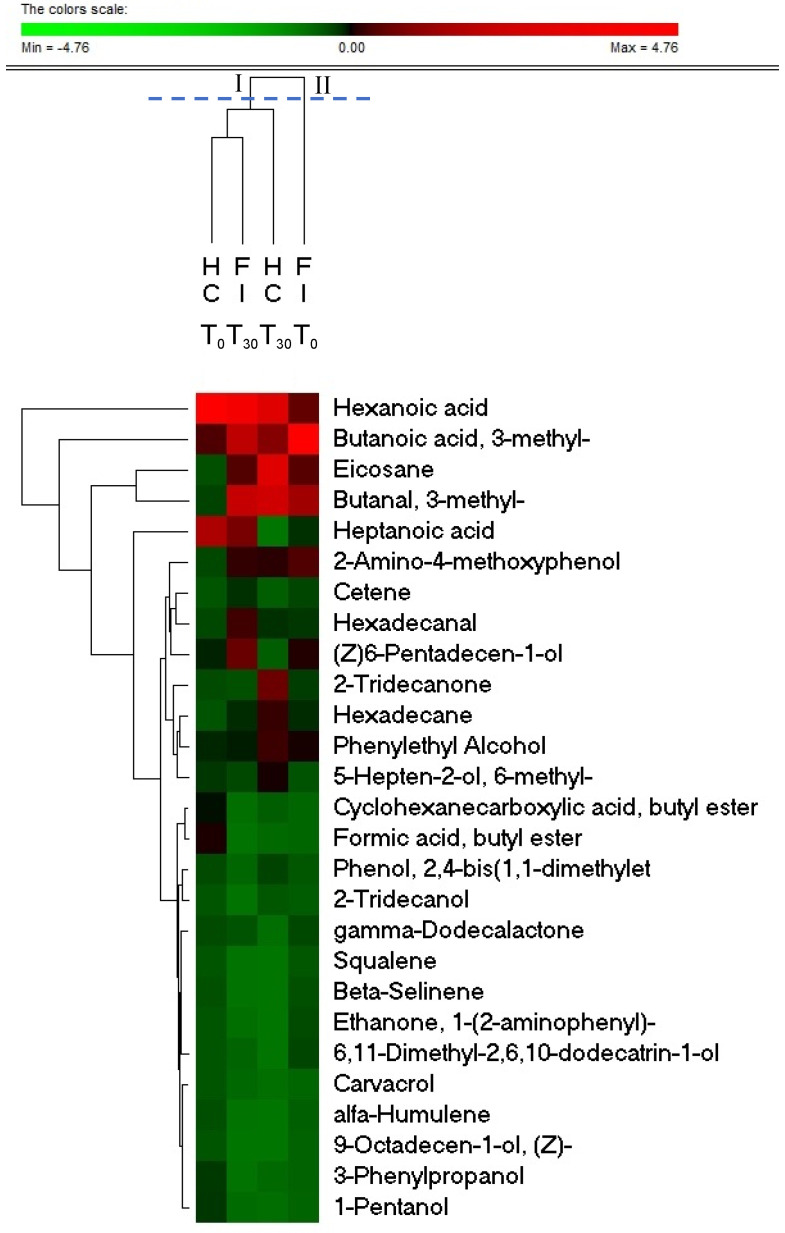
Permutation analyses of significantly different VOCs evaluated by comparing Healthy Control (HC) and Fructose-Intolerant (FI) subjects before (T0) and after the treatment (T30).

The statistically significant differences in compounds were used for the subsequent permutation analysis (Figure 4). These analyses clearly showed how the VOCs profile of HC-T0, HC-T30, and FI-T30 were grouped in one single cluster (cluster I), whereas the fructose-intolerant group at the baseline (FI-T0) was un-clustered. The cluster I was mainly characterized by a lower amount of 3-methyl-butanoic acid and a higher amount of hexanoic acid compared with FI-T0.

### 3.5. Correlation between Clinical Symptoms and VOCs

**Figure 5:**
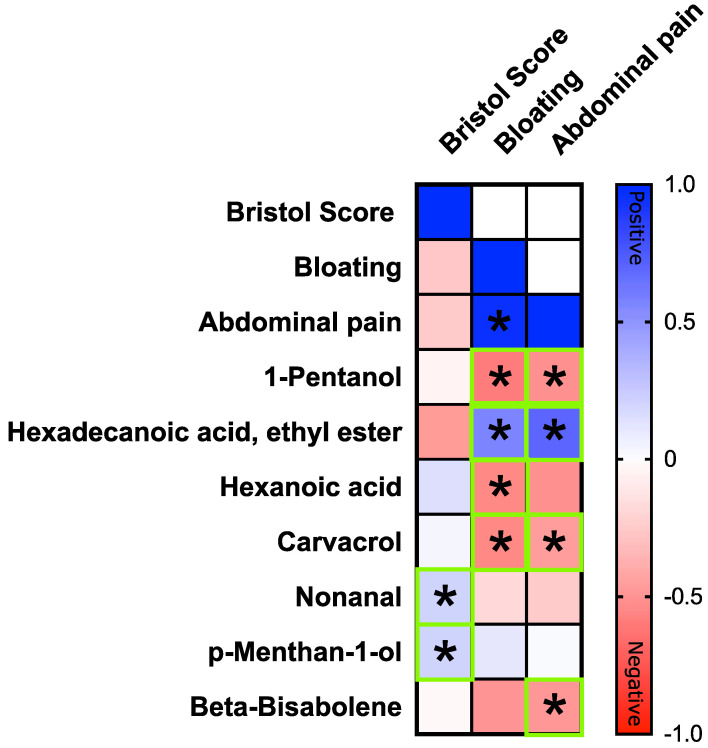
Spearman correlation on FI patients between VOCs (ppm) and clinical symptoms (Visual Analogue Scale 0–100). The scaled matrices were merged and used for the correlation computing. Only statistically significant correlations (*p* < 0.05) were plotted and marked with asterisk (*). The color graduated scale ranges from −1 (red: negative correlations) to 1 (blue: positive correlations).

A Spearman’s correlation analysis was used to determine the relationships between clinical symptoms and VOCs amount and only statistically significant correlations (*p* < 0.05) were shown (Figure 5). The bloating scores in fructose-intolerant subjects was negatively correlated with 1-pentanol (r = −0.599, *p*-value = 0.0343), hexanoic acid (r = −0.534, *p*-value = 0.0412), and carvacrol (r = −0.589, *p*-value = 0.0302). Interestingly, the abdominal pain score showed the same trend, in particular, a negative correlation was assessed with 1-pentanol (r = −0.503, *p*-value < 0.05), carvacrol, and beta-bisabolene (r = −0.500, *p*-value < 0.05). Conversely, the ethyl ester of hexadecenoic acid was positively correlated with bloating (r = 0.544, *p*-value < 0.05) and abdominal pain (r = 0.601, *p*-value = 0.01) scores.

## 4. Discussion

In this study, we profiled a Romanian cohort of patients affected by Functional Gastrointestinal Disorders (FGIDs) compared with healthy controls (HC) characterized by the occurrence of fructose intolerance. The effects of a specific probiotic containing *L. plantarum* strains (CECT 7484 and CECT 7485), and *P. acidilactici* CECT 7483 in both groups led us to obtain interesting results in terms of major gastrointestinal symptom improvement and metabolomic profile changes. We observed distinct outcomes in fructose intolerant patients when compared with healthy controls after one month of treatment.

As first remark, we observed that within our specific Romanian cohort, the percentage of fructose intolerants (31.8%), within the FGID group, was higher than that of lactose intolerants (6.8%). In a large cohort of North European and Mediterranean Caucasian population, a higher prevalence of fructose intolerance in FGIDs was reported [8]. Barrett et al. [7] evaluated the prevalence of fructose and lactose malabsorption in healthy subjects and patients affected by chronic intestinal gastrointestinal disorders (Crohn’s disease, ulcerative colitis, coeliac disease, FGIDs). More in detail, a higher percentage of fructose malabsorption was found in subjects affected by Crohn’s disease compared with the other groups [7]. Regarding the FGID group, the prevalence of fructose malabsorption was higher than lactose one and was specifically observed in patients with IBS constipation-predominant, IBS diarrhea-predominant, functional bloating, functional constipation, and diarrhea. All these cited findings are in line with our results stating that the occurrence of food intolerance in FGIDs could be mostly related to fructose.

Regardless the symptoms, we observed that the two subject groups did not show any significant difference at baseline, i.e., age, sex, body mass index (BMI), and bowel habits. Although the number of overweight subjects can be considered a limitation of the present study, the prevalence of subjects over the normal range limit (24.9 kg/m^2^) in HC and FI groups was equally distributed. Concerning bowel habits, we observed that both groups showed averaged Bristol score values fitting the normal range (i.e., 3–4). Noteworthy, in FI subjects we observed a higher prevalence of constipation than diarrheic status. Besides our results, fructose intolerance is usually correlated with high Bristol score values (diarrhea score 5–7) due to the osmotic load that results from the unabsorbed fructose into the intestinal lumen [9]. Interestingly, in line with us, Barret et al. [7] reported how the 44% of patients with functional constipation and the 55% of patients with constipation-predominant IBS both exhibited fructose malabsorption.

With this in mind, we evaluated the effectiveness of a probiotic EQBIOTA formulation in improving FI related gastrointestinal symptoms, whose occurrence is mainly due to a suboptimal fluid balance between blood and the intestinal lumen, as well as to the fermentation of unabsorbed fructose. These result in the production of compounds (i.e., hydrogen, methane, carbon dioxide, SCFA, and other gases) which can cause bloating, flatulence, and abdominal pain [9]. After 30 days of treatment with EQBIOTA, bloating and abdominal pain decreased significantly in our patients. In a previous study on lactose-intolerant patients, we demonstrated that probiotics *Bifidobacterium longum* BB536 and *Lactobacillus rhamnosus* HN001 plus vitamin B6 ameliorated common functional gastrointestinal symptoms such as abdominal pain, bloating, bowel habits, fecal microbiota, and related metabolome [28].

An additional aspect to be considered is that changes in intestinal fructose absorption and luminal concentrations of dietary fructose can profoundly affect the gut microbiota with the acquisition of a microbiome with altered metabolic capacity [42]. Greater levels of *Clostridium* and *Enterococcus* spp. have been reported in mice undergoing targeted GLUT2 deletion with an overexpression of GLUT5 [43]. In addition, a high fructose intake can increase intestinal bacterial load [44] and composition of the human gut microbiome [8,45]. Studies on animal models revealed how fructose ingestion increased the numbers of Gram-negative bacteria in mice [44], whereas Gram-positive anaerobic bacteria increased in rats [46,47,48]. In light of this, we cannot exclude that the changes of bacterial load and composition in our cohort occurred or can be influenced by changes in fructose intake and absorption.

Significant differences in VOC profile were evaluated between FI and HC groups at baseline, whereas the EQBIOTA treatment reduced this divergence. Indeed, our analyses showed how the evaluated groups were divided in two clusters HC-T0, HC-T30, and FI-T30 on one side and FI-T0 on the other one (Figure 4). In the FI-T30 group, Medium Chain Fatty Acids (MCFAs) resulted as higher compared with FI-T0, specifically hexanoic acid and heptanoic acid. Interestingly, the MCFAs resulted in distinguishing healthy subjects and patients with gastrointestinal pathologies. De Preter et al. [49] reported a higher value of hexanoic acid in a control group compared with patients with Inflammatory Bowel Disease. Hexanoic acid also exerted a potential positive role as immune system modulator in the regulation of Interleukin-32, an important cytokine involved in inflammation and cancer development [50]. These data are also in line with our findings, which reported a negative correlation between hexanoic acid and the worsening of gastrointestinal clinical symptoms. Moreover, our results described a reduction of isovaleric acid (butanoic acid, 3-methyl) after treatment. The improvement of slow transit constipation has been correlated with the reduction of isovaleric acid levels after treatment with Astragaloside IV in mouse model [51]. Thus, we can hypothesize that the used formulation improves symptoms with a decrease in isovaleric acid levels that could act on slow transit constipation.

A positive correlation between gastrointestinal clinical symptoms and ethyl ester of hexadecanoic acid, a compound belonging to the Fatty Acid Ethyl Esters (FAEEs) was also observed. It has been reported that FAEEs are able to induce dysfunctions of the intestinal barrier, through the induction of cellular oxidative stress of the intestinal *epithelium* [52]. In this case, a higher level of this ethyl ester is thought to be coupled with symptom worsening. Importantly, we detected an increase of carvacrol in FI group after treatment and a negative correlation with gastrointestinal symptoms. Several studies reported the beneficial effects of carvacrol, used as treatment for different pathologies. In detail, carvacrol showed a potential role as a gastroprotective agent against gastric lesions in rodents [53]. Moreover, carvacrol could be used to counteract dysbiosis and to reduce *Clostridium difficile* infection [54]. It was also reported that carvacrol had a protective effect on mice from acetic acid-induced colitis, by reducing inflammatory, nociceptive, and oxidative damage [55]. In vivo studies have shown that the EQBIOTA probiotic formula displays a protective effect on animal models of experimental colitis [56], as well effectiveness in cholesterol lowering and alleviation of allergic reactions [57,58]. Further, this probiotic combination strains have proven effectiveness in improving the symptomatology for patient with IBS [59,60]. The well-known beneficial effects of probiotic formulation of *L. plantarum* and *P. acidilactici* to repair gut barrier functions, balance altered microbiota, and restore local and systemic immune regulation could explain the main findings of our study that suggest a positive beneficial effect of this probiotic on FI subjects.

## 5. Conclusions

With the aim to ascertain an improvement in persistent functional gastrointestinal symptoms, we here administered a novel probiotic formulation (*Lactiplantibacillus plantarum* CECT 7484 and CECT 7485 and *Pediococcus acidilactici* CECT 7483) to a FI Romanian cohort of patients that were under a 30-days free-fructose diet. The treatment determined changes in VOC profile with a specific increase of potentially anti-inflammatory and protective compounds. These changes occured in parallel with a decrease of Fatty Acid Ethyl Esters, which might have a potential detrimental effect on the intestinal barrier and oxidative stress. Our preliminary observations suggest that such metabolomic variations occur with improvement of gastrointestinal symptoms and require further observations in larger cohorts from different geographical areas.

A limitation of this study is the small number of enrolled subjects. Although we started by gathering a high number of FGID patients, the exclusion criteria seriously limited the final analyzed cohort size.

Due to this restriction, the viability of the study relies in fact on the comparison of fructose-intolerant patients with a healthy control parallel arm. We used metabolomic analyses and symptom improvement with the aim of documenting the efficacy of the novel probiotic consumption for one month.

## Figures and Tables

**Table 1:** Baseline characteristics expressed as mean ± SD and median values of the fructose intolerant and healthy control group enrolled for EQBIOTA treatment.

	Healthy Control	Fructose Intolerant	*p*-Value
Number	13	14	-
Age (years)	54.0 ± 16.5 (56.0)	48.7 ± 15.5 (49.5)	n.s.
Males: Females	7:6	6:8	n.s.
BMI (Kg/m^2^)	28.4 ± 5.9 (28.7)	25.7 ± 5.2 (24.8)	n.s.
Bristol score (BSFS)	3.3 ± 0.5 (3.0)	2.9 ± 1.1 (3.0)	n.s.
Bloating (VAS, mm)	6.9 ± 11.8 (0.0)	68.6 ± 21.4 (70.0)	*p* = 0.0001
Abdominal pain (VAS, mm)	2.3 ± 4.4 (0.0)	43.6 ± 28.7 (35.0)	*p* = 0.0001

Data are mean ± SD and (median). Statistics: Two-tailed unpaired *t*-tests corrected with Holm–Sidak multiple comparison. Abbreviations: n.s., not significant; BMI, Body Mass Index; BSFS, Bristol Stool Form Scale; VAS, Visual Analogue Scale.

**Table 2:** Composition of the 30-day diet as micro- and macro-nutrients in the study groups prior to EQBIOTA treatment.

Nutrient	Healthy Control	Fructose Intolerant	*p*-Value
Fiber (g)	7.2 ± 1.0	7.1 ± 1.1	n. s.
Carbohydrates (%)	47.0 ± 3.5	45.7 ± 3.8	n. s.
Proteins (%)	19.3 ± 1.2	18.9 ± 1.3	n. s.
Animal proteins/tot (%)	63.9 ± 5.2	64.8 ± 5.9	n. s.
Vegetable proteins/tot (%)	36.1 ± 5.2	35.2 ± 5.9	n. s.
Lipids (%)	34.9 ± 2.6	35.1 ± 2.8	n. s.
Saturated fatty acids/tot (%)	40.8 ± 3.5	41.4 ± 3.6	n. s.
Vitamin B6 (mg)	1.1 ± 0.2	1.2 ± 0.2	n. s.

Data are mean ± SD; n.s., not significant.

**Table 3:** Bowel habits and gastro-intestinal symptoms observed in the fructose-intolerant and healthy control groups at the baseline (T0) and after 30 days of EQBIOTA treatment (T30).

	Healthy Control			Fructose Intolerant		
	T0	T30	*p*-Value	T0	T30	*p*-Value
Bristol Score (BSFS)	3.3 ± 0.5	3.6 ± 0.4	n.s.	2.9 ± 1.1	3.4 ± 0.5	n.s.
Bloating (VAS, mm)	6.9 ± 11.8	11.2 ± 6.5	n.s.	68.6 ± 21.4	13.6 ± 17.8	*p* = 0.0001
Abdominal pain (VAS, mm)	2.3 ± 4.4	5.9 ± 3.1	n.s.	43.6 ± 28.7	7.1 ± 13.3	*p* = 0.0002

Data are mean ± SD. Statistics: Two-tailed unpaired *t*-tests corrected with Holm–Sidak multiple comparison. Abbreviations: T0, baseline; T30, after 30 days of treatment; n.s., not significant; BSFS, Bristol Stool Form Scale; VAS, Visual Analogue Scale.

**Table 4:** Relative concentration (expressed as µg/g of internal standard ± SD) of volatile organic compounds (VOCs) in the fructose-intolerant (FI) and healthy control (HC) groups at the baseline (T0) and after 30 days of EQBIOTA treatment (T30). Only statistically significant differences (*p*-value < 0.05) are reported.

Compounds	FI_T0_	FI_T30_	HC_T0_	HC_T30_	FI_T0_ vs. FI_T30_	HC_T0_ vs. HC_T30_	HC_T0_ vs. FI_T0_	HC_T30_ vs. FI_T30_
	µg/g	µg/g	µg/g	µg/g	*p*-value *	*p*-value	*p*-value	*p*-value
1-Pentanol	0.02 ± 0.045	0.1 ± 0.121	0.23 ± 0.324	0.05 ± 0.096	0.048	n.s.	0.044	n.s.
Sulcatol (5-Hepten-2-ol, 6-methyl-)	0.12 ± 0.232	0.26 ± 0.193	0.24 ± 0.409	0.48 ± 0.4	0.019	n.s.	n.s.	n.s.
9-Octadecen-1-ol, (Z)-	0.03 ± 0.055	0.04 ± 0.043	n.d.	n.d.	n.s.	n.s.	n.s.	0.01
Phenylethyl Alcohol	0.39 ± 0.297	0.4 ± 0.478	0.32 ± 0.288	0.59 ± 0.456	n.s.	0.028	n.s.	n.s.
2-Tridecanol	0.07 ± 0.157	0.05 ± 0.138	n.d.	0.19 ± 0.15	n.s.	n.s.	n.s.	0.041
3-Phenylpropanol	0.03 ± 0.08	0.06 ± 0.096	0.2 ± 0.184	0.08 ± 0.141	n.s.	n.s.	0.031	n.s.
(Z)6-Pentadecen-1-ol	0.41 ± 0.439	0.73 ± 0.658	0.34 ± 0.486	0.16 ± 0.138	n.s.	n.s.	n.s.	0.022
Butanal, 3-methyl-	1.26 ± 0.812	1.57 ± 1.48	0.14 ± 0.246	2 ± 1.828	n.s.	n.s.	0.027	n.s.
Hexadecanal	0.25 ± 0.134	0.54 ± 0.455	0.12 ± 0.207	0.37 ± 0.199	0.039	n.s.	n.s.	n.s.
Formic acid, butyl ester	n.d.	0.06 ± 0.096	0.46 ± 0.64	0.08 ± 0.224	n.s.	n.s.	0.035	n.s.
Cyclohexanecarboxylic acid, butyl ester	n.d.	0.07 ± 0.186	0.4 ± 0.421	0.15 ± 0.267	n.s.	n.s.	0.013	n.s.
6,11-Dimethyl-2,6,10-dodecatrin-1-ol)	0.18 ± 0.314	0.14 ± 0.179	n.d.	n.d.	n.s.	n.s.	n.s.	0.032
Hexadecane	0.29 ± 0.326	0.36 ± 0.361	0.03 ± 0.055	0.56 ± 0.447	0.018	n.s.	n.s.	n.s.
Cetene	0.18 ± 0.132	0.35 ± 0.312	n.d.	0.15 ± 0.125	n.s.	n.s.	0.024	n.s.
Eicosane	0.63 ± 1.241	0.61 ± 1.437	0.05 ± 0.051	2.35 ± 1.902	n.s.	n.s.	n.s.	0.039
2-Amino-4-methoxyphenol	0.59 ± 0.223	0.5 ± 0.388	0.1 ± 0.108	0.53 ± 0.26	n.s.	n.s.	0.004	n.s.
gamma-Dodecalactone	0.17 ± 0.148	0.22 ± 0.241	0.09 ± 0.117	0.06 ± 0.044	n.s.	n.s.	n.s.	0.048
2-Tridecanone	0.23 ± 0.45	0.23 ± 0.476	0.09 ± 0.104	0.84 ± 0.657	n.s.	n.s.	n.s.	0.036
Ethanone, 1-(2-aminophenyl)-	0.16 ± 0.318	0.08 ± 0.106	n.d.	n.d.	n.s.	n.s.	n.s.	0.043
Butanoic acid, 3-methyl-	3.63 ± 3.634	1.46 ± 1.984	0.75 ± 0.791	1.02 ± 1.618	0.043	n.s.	n.s.	n.s.
Hexanoic acid	0.7 ± 1.122	2.52 ± 2.944	3.84 ± 5.948	2.29 ± 0.62	0.037	n.s.	n.s.	n.s.
Heptanoic acid	0.27 ± 0.475	0.82 ± 1.124	1.99 ± 3.165	n.d.	0.046	n.s.	n.s.	0.039
Phenol, 2,4-bis(1,1-dimethylethyl)	0.1 ± 0.086	0.13 ± 0.081	0.09 ± 0.088	0.29 ± 0.213	n.s.	n.s.	n.s.	0.04
alfa-Humulene	0.05 ± 0.085	0.05 ± 0.065	0.07 ± 0.114	n.d.	n.s.	n.s.	n.s.	0.04
Beta-Selinene	0.13 ± 0.164	0.06 ± 0.073	0.05 ± 0.09	n.d.	n.s.	n.s.	n.s.	0.029
Squalene	0.1 ± 0.142	0.05 ± 0.093	n.d.	n.d.	0.043	n.s.	n.s.	n.s.
Carvacrol	n.d.	0.12 ± 0.163	n.d.	0.04 ± 0.115	0.048	n.s.	n.s.	n.s.

n.d. not detected; n.s. not significant. * Only statistically significant differences (Wilcoxon test) in at least one comparison have been reported.

## Data Availability

Not applicable.

## Supplementary Materials

The following supporting information can be downloaded at: https://www.mdpi.com/article/10.3390/nu14122488/s1, Table S1: Concentration of volatile organic (µg/g of internal standard) compounds (identified in fecal samples of fructose intolerant subjects (FI) and healthy control (HC) at the baseline (T0) and after 30 days of treatment (T30) with EQBIOTA.
