# Supplementary material for: Clinical and Metabolomic Effects of Lactiplantibacillus plantarum and Pediococcus acidilactici in Fructose Intolerant Patients
Source: Nutrients. 2022 Jun 15;14(12):2488. doi: 10.3390/nu14122488 (PMC9231202; doi:10.3390/nu14122488)
Supplement: Supplementary file 1 [file nutrients-14-02488-s001.zip › nutrients-1744861-supplementary.pdf]

Supplementary Table S1: Concentration of volatile organic (µg/g of internal standard) compounds (identified in faecal samples of fructose intolerant subjects (FI) and healthy control (HC) at the baseline (T0) and after 30 days of treatment (T30) with EQBIOTA.

Compounds	Groups	Basaline (T0)	Treatment (T30)
Alcohols			
Ethanol	HC	0.09(0.04, 0.17)	0.16(0.15, 0.24)
	FI	0.2(0.09, 0.23)	0.1(0.06, 0.21)
1-Butanol, 3-methyl-	HC	n.d.	0.74(0, 1.46)
	FI	0.21(0, 0.23)	0(0, 0.19)
1-Pentanol	HC	0.09(0.05, 0.35)	0(0, 0.07)
	FI	n.d.	0(0, 0.2)
1-Hexanol	HC	0(0, 1.09)	0.17(0.08, 0.22)
	FI	0(0, 0.06)	0.15(0, 0.24)
Sulcatol (5-Hepten-2-ol, 6-methyl-)	HC	0(0, 0.35)	0.49(0.14, 0.78)
	FI	0(0, 0.1)	0.29(0, 0.4)
2-Undecanol	HC	0(0, 0.22)	0.88(0, 1.13)
	FI	n.d.	0(0, 0.11)
1-Undecanol	HC	n.d.	0(0, 0.11)
	FI	n.d.	n.d.
Benzyl alcohol	HC	0.09(0.04, 0.14)	0(0, 0.12)
	FI	0(0, 0.04)	0(0, 0.12)
9-Octadecen-1-ol, (Z)-	HC	n.d.	n.d.
	FI	0(0, 0.05)	0.06(0, 0.07)
Phenylethyl Alcohol	HC	0.23(0.15, 0.43)	0.54(0.26, 0.73)
	FI	0.36(0.2, 0.5)	0.35(0.07, 0.45)
2-Tridecanol	HC	n.d.	0.2(0.09, 0.27)
	FI	0(0, 0.04)	n.d.
3-Phenylpropanol	HC	0.25(0.13, 0.31)	0(0, 0.12)
	FI	n.d.	0(0, 0.1)
1-Hexadecanol	HC	0.62(0.43, 0.72)	0.66(0.62, 1.14)
	FI	1.39(0.67, 1.67)	0.87(0.81, 1.3)
(Z)6-Pentadecen-1-ol	HC	0.07(0.06, 0.48)	0.11(0.07, 0.23)
	FI	0.12(0.07, 0.7)	0.54(0.24, 0.94)
2-Propyl-1-pentanol	HC	0(0, 0.16)	n.d.
	FI	n.d.	n.d.
Aldehydes			
Butanal, 3-methyl-	HC	0(0, 0.21)	1.66(0.38, 3.73)
	FI	1.19(0.73, 1.65)	1.23(0.63, 1.8)
Nonanal	HC	0.07(0.04, 0.12)	0.51(0.28, 0.58)
	FI	0.12(0.06, 0.21)	0.19(0.15, 0.31)
Benzaldehyde	HC	0.87(0.54, 3.25)	0.96(0.77, 1.99)
	FI	0.97(0.51, 1.6)	0.76(0.27, 1.87)
Hexadecanal	HC	0(0, 0.18)	0.31(0.27, 0.48)
	FI	0.32(0.2, 0.35)	0.33(0.27, 0.71)
Esters			

Methyl propionate	HC	n.d.	n.d.
	FI	n.d.	n.d.
Ethyl Acetate	HC	n.d.	n.d.
	FI	0(0, 0.25)	0(0, 0.04)
Propanoic acid, ethyl ester	HC	n.d.	n.d.
	FI	0(0, 0.21)	0(0, 0.07)
n-Propyl acetate	HC	n.d.	n.d.
	FI	0(0, 0.11)	n.d.
Butanoic acid, methyl ester	HC	0(0, 0.07)	n.d.
	FI	0(0, 0.07)	n.d.
Butanoic acid, ethyl ester	HC	0(0, 0.04)	0(0, 0.14)
	FI	0(0, 0.69)	0.14(0.07, 0.24)
Propanoic acid, propyl ester	HC	n.d.	n.d.
	FI	0(0, 0.25)	0(0, 0.1)
Butanoic acid, 3-methyl-, ethyl ester	HC	n.d.	n.d.
	FI	0(0, 0.03)	0(0, 0.06)
Acetic acid, butyl ester	HC	n.d.	n.d.
	FI	n.d.	n.d.
Methyl valerate	HC	0(0, 0.07)	n.d.
	FI	n.d.	n.d.
Butanoic acid, propyl ester	HC	0(0, 0.09)	0(0, 0.03)
	FI	0(0, 0.65)	0(0, 0.12)
Pentanoic acid, ethyl ester	HC	n.d.	0(0, 0.08)
	FI	0(0, 0.11)	0.15(0, 0.17)
Propanoic acid, butyl ester	HC	0(0, 0.07)	n.d.
	FI	n.d.	n.d.
Butanoic acid, 3-methyl-, propyl ester	HC	n.d.	n.d.
	FI	n.d.	n.d.
Formic acid, butyl ester	HC	0.19(0.1, 0.69)	n.d.
	FI	n.d.	0(0, 0.09)
Butanoic acid, butyl ester	HC	0(0, 0.39)	n.d.
	FI	0(0, 0.03)	n.d.
Pentanoic acid, propyl ester	HC	n.d.	n.d.
	FI	0(0, 0.22)	n.d.
Hexanoic acid, ethyl ester	HC	0(0, 0.16)	n.d.
	FI	n.d.	0(0, 0.1)
Pentanoic acid, butyl ester	HC	0(0, 0.11)	0(0, 0.06)
	FI	n.d.	n.d.
Butanoic acid, pentyl ester	HC	0(0, 0.07)	n.d.
	FI	n.d.	n.d.
Hexanoic acid, propyl ester	HC	0(0, 0.24)	n.d.
	FI	n.d.	n.d.
Cyclohexanecarboxylic acid, methyl ester	HC	n.d.	n.d.
	FI	0(0, 0.08)	n.d.
Cyclohexanecarboxylic acid, ethyl ester	HC	0.2(0.1, 0.26)	0(0, 0.43)

	FI	0(0, 2.05)	n.d.
Cyclohexanecarboxylic acid, propyl ester	HC	0.43(0.22, 0.46)	0(0, 0.23)
	FI	0(0, 0.62)	n.d.
Decanoic acid, methyl ester	HC	n.d.	n.d.
	FI	n.d.	n.d.
Cyclohexanecarboxylic acid, butyl ester	HC	0.35(0.17, 0.59)	0(0, 0.22)
	FI	n.d.	n.d.
Decanoic acid, ethyl ester	HC	n.d.	n.d.
	FI	n.d.	n.d.
Ethyl salicylate OR Methyl salicylate	HC	0(0, 0.06)	n.d.
	FI	n.d.	n.d.
Benzeneacetic acid, ethyl ester	HC	n.d.	n.d.
	FI	0(0, 0.03)	n.d.
Dodecanoic acid, methyl ester	HC	n.d.	0(0, 0.06)
	FI	n.d.	n.d.
Dodecanoic acid, ethyl ester	HC	n.d.	0.57(0, 0.77)
	FI	n.d.	n.d.
Benzenepropanoic acid, ethyl ester	HC	0.06(0.03, 0.08)	n.d.
	FI	n.d.	0(0, 0.04)
Dodecanoic acid, propyl ester	HC	n.d.	n.d.
	FI	n.d.	n.d.
Methyl tetradecanoate	HC	n.d.	n.d.
	FI	n.d.	n.d.
Hexadecanoic acid, methyl ester	HC	n.d.	0(0, 0.03)
	FI	n.d.	n.d.
Hexadecanoic acid, ethyl ester	HC	0(0, 0.04)	0(0, 0.19)
	FI	0(0, 0.04)	n.d.
Hydrocarbons			
Ethylamine	HC	0.33(0.28, 0.56)	1.34(0.48, 1.5)
	FI	0.67(0.29, 0.89)	0.62(0.38, 1.26)
Decane, 4-methyl	HC	n.d.	n.d.
	FI	0(0, 0.03)	0(0, 0.02)
Benzene, 1,3-bis(1,1-dimethylethyl)	HC	0(0, 0.36)	0.27(0, 0.43)
	FI	0(0, 1.94)	0.32(0.24, 2.87)
Methane, di-tert-butoxy-	HC	0.3(0.18, 0.36)	0.38(0.28, 0.54)
	FI	0.26(0.24, 0.67)	0.32(0.3, 0.82)
Pentadecane	HC	0.11(0.05, 0.12)	0.64(0.25, 0.86)
	FI	0.2(0.08, 0.26)	0.1(0.05, 0.25)
3,5-Heptadien-2-one, 6-methyl-	HC	0.23(0.12, 0.77)	0(0, 0.34)
	FI	n.d.	0.64(0, 1.11)
γ-Pentalactone	HC	n.d.	n.d.
	FI	n.d.	n.d.
6,11-Dimethyl-2,6,10-dodecatrin-1-ol	HC	n.d.	n.d.
	FI	0(0, 0.26)	0(0, 0.28)
Hexadecane	HC	0(0, 0.05)	0.6(0.24, 0.84)

	FI	0.21(0.11, 0.31)	0.29(0.17, 0.35)
D-Limonene	HC	0(0, 0.6)	0.27(0.11, 0.46)
	FI	0.33(0.19, 0.59)	0.54(0.22, 0.83)
Estragole	HC	n.d.	n.d.
	FI	n.d.	n.d.
Cetene	HC	n.d.	0.16(0.07, 0.23)
	FI	0.23(0.11, 0.26)	0.28(0.14, 0.55)
2,4-Decadienal	HC	0(0, 0.08)	n.d.
	FI	n.d.	n.d.
Eicosane	HC	0.04(0.02, 0.07)	2.57(1.04, 3.37)
	FI	0.12(0.08, 0.35)	0(0, 0.22)
2-Hexadecene, 3,7,11,15-tetramethyl	HC	n.d.	n.d.
	FI	0(0, 0.06)	0(0, 0.1)
Benzene, (1-ethyl-1-propenyl)-	HC	0(0, 0.02)	0.35(0.1, 0.79)
	FI	0.06(0, 0.24)	0(0, 0.17)
Propane, 1-isothiocyanato-3-(methylthio)-	HC	n.d.	n.d.
	FI	n.d.	0(0, 0.34)
gamma-n-Amylbutyrolactone	HC	n.d.	n.d.
	FI	n.d.	n.d.
2-Amino-4-methoxyphenol	HC	0.09(0.04, 0.15)	0.53(0.35, 0.59)
	FI	0.67(0.42, 0.76)	0.66(0.14, 0.69)
gamma-Dodecalactone	HC	0.04(0.02, 0.13)	0.06(0.02, 0.09)
	FI	0.12(0.06, 0.27)	0.15(0.06, 0.35)
Indoles			
Indole	HC	10.93(9.24, 16.71)	16.28(13.21, 20.43)
	FI	18.62(7.39, 49.7)	21.63(6.85, 33.09)
1H-Indole, 3-methyl-	HC	0.63(0.48, 3.29)	23.06(12.1, 27.76)
	FI	1.42(0.69, 14.19)	0.58(0.3, 8.94)
Ketones			
Sulcatone	HC	1.03(0.64, 5.27)	2.17(1.99, 2.67)
	FI	0.85(0.45, 2.29)	5.34(0.94, 7.46)
2-Nonanone	HC	0(0, 0.05)	0(0, 0.38)
	FI	n.d.	n.d.
2-Undecanone	HC	0(0, 0.32)	1.54(0.49, 2.26)
	FI	0.39(0.12, 0.51)	0(0, 0.4)
Benzyl methyl ketone	HC	0(0, 0.03)	n.d.
	FI	0(0, 0.07)	n.d.
2-Tridecanone	HC	0.06(0.03, 0.13)	0.87(0.38, 1.24)
	FI	0(0, 0.2)	0(0, 0.16)
2-Tetradecanone	HC	n.d.	0(0, 0.08)
	FI	0(0, 0.08)	n.d.
2-Pentadecanone	HC	0(0, 0.31)	n.d.
	FI	n.d.	n.d.
2-Piperidinone	HC	n.d.	0(0, 0.14)
	FI	0(0, 0.09)	0(0, 0.05)

Ethanone, 1-(2-aminophenyl)-	HC	n.d.	n.d.
	FI	0(0, 0.13)	0(0, 0.14)
Organic acids			
Acetic acid	HC	0.26(0.13, 1.03)	0.38(0.09, 0.93)
	FI	0.34(0.2, 0.67)	0.33(0.09, 1.3)
Propanoic acid	HC	0.61(0.3, 1.12)	0(0, 1.28)
	FI	0.73(0.61, 0.93)	0.49(0.11, 2.27)
Propanoic acid, 2-methyl-	HC	0.09(0.09, 0.21)	0(0, 0.35)
	FI	0.47(0.1, 0.64)	0.11(0, 0.53)
Butanoic acid	HC	3.9(2.31, 7.47)	1.6(1.03, 7.16)
	FI	2.86(1.95, 3.52)	1.8(1.02, 8.57)
Butanoic acid, 3-methyl-	HC	0.67(0.34, 1.12)	0(0, 1.52)
	FI	2.7(0.77, 5.98)	0.21(0, 2.45)
Pentanoic acid	HC	2.18(1.4, 4.25)	0(0, 4.42)
	FI	2.53(0.43, 3.54)	0(0, 7.71)
Pentanoic acid, 4-methyl-	HC	0(0, 0.07)	0(0, 0.05)
	FI	0(0, 0.26)	n.d.
Hexanoic acid	HC	0.83(0.41, 5.76)	2.27(2.06, 2.62)
	FI	0(0, 1.03)	1.81(0.25, 3.64)
Heptanoic acid	HC	0.34(0.17, 2.99)	n.d.
	FI	0(0, 0.39)	0.2(0, 1.32)
Octanoic acid	HC	0.07(0.04, 0.37)	0(0, 0.48)
	FI	0(0, 0.04)	0(0, 0.1)
Nonanoic acid	HC	0.22(0.21, 1.34)	0.41(0.05, 1)
	FI	0.31(0.08, 0.79)	0.18(0, 1.1)
Phenols			
Phenol	HC	0.18(0.17, 0.26)	1.65(0.74, 2.01)
	FI	0.74(0.2, 4.63)	0.82(0.29, 1.41)
4-methylphenol (p-cresol)	HC	26.63(24.81, 30.7)	105.18(58.94, 115)
	FI	72.37(37.5, 80.59)	44.8(17.05, 75.79)
Phenol, 4-ethyl-	HC	n.d.	n.d.
	FI	0(0, 0.1)	n.d.
2-Methoxy-4-vinylphenol	HC	0(0, 0.05)	0.09(0.03, 0.15)
	FI	0.06(0, 0.1)	0.06(0, 0.11)
Phenol, 2,4-bis(1,1-dimethylet	HC	0.06(0.04, 0.12)	0.3(0.12, 0.39)
	FI	0.09(0.05, 0.12)	0.15(0.09, 0.16)
Sulfur Compounds			
Disulfide, dimethyl	HC	0.06(0.03, 0.16)	0.55(0.23, 0.93)
	FI	0.15(0.14, 0.43)	0.15(0, 0.22)
Dimethyl trisulfide	HC	0.1(0.05, 0.12)	0.24(0, 0.4)
	FI	0.1(0, 0.13)	0(0, 0.51)
Terpenes			
3-Carene	HC	n.d.	n.d.
	FI	0(0, 0.05)	0(0, 0.04)
gamma-Terpinene	HC	n.d.	n.d.

	FI	n.d.	n.d.
Caparratriene	HC	n.d.	0.37(0.15, 0.64)
	FI	0(0, 0.07)	n.d.
D- Linalol	HC	0(0, 0.12)	n.d.
	FI	0(0, 0.07)	0(0, 0.07)
beta-Caryophyllene	HC	0.16(0.08, 1.52)	1.82(1.02, 2.23)
	FI	0.21(0, 2.37)	0.74(0.55, 2.27)
p-Menthan-1-ol	HC	0.81(0.6, 4.15)	0(0, 0.09)
	FI	0.38(0.03, 1.11)	0.35(0.22, 0.4)
alfa-Humulene	HC	0(0, 0.1)	n.d.
	FI	0(0, 0.07)	0(0, 0.09)
Beta-Selinene	HC	0(0, 0.08)	n.d.
	FI	0.08(0, 0.2)	0(0, 0.12)
Beta-Bisabolene	HC	0(0, 0.04)	0.11(0, 0.16)
	FI	n.d.	0(0, 0.25)
Citral	HC	0(0, 0.23)	0.1(0.08, 0.12)
	FI	0(0, 0.06)	0.17(0, 0.2)
alfa-Farnesene	HC	n.d.	n.d.
	FI	0.2(0, 0.44)	0.1(0, 0.76)
Geranyl acetone	HC	0.2(0.1, 0.23)	0(0, 0.29)
	FI	0.2(0.17, 0.25)	0.21(0.1, 0.42)
Squalene	HC	n.d.	n.d.
	FI	0(0, 0.18)	0(0, 0.09)
Carvacrol	HC	n.d.	n.d.
	FI	n.d.	0(0, 0.22)

n.d. , not detected

Data are the median values (Q1, Q3) of relative concentration (expressed as μg/g of internal standard)
